# The Pathologic Effect of a Novel Neomorphic *Fgf9^Y162C^* Allele Is Restricted to Decreased Vision and Retarded Lens Growth

**DOI:** 10.1371/journal.pone.0023678

**Published:** 2011-08-17

**Authors:** Oliver Puk, Gabriele Möller, Arie Geerlof, Kathrin Krowiorz, Nafees Ahmad, Sibylle Wagner, Jerzy Adamski, Martin Hrabé de Angelis, Jochen Graw

**Affiliations:** 1 Helmholtz Center Munich, German Research Center for Environmental Health, Institute of Developmental Genetics, Neuherberg, Germany; 2 Helmholtz Center Munich, German Research Center for Environmental Health, Institute of Experimental Genetics, Neuherberg, Germany; 3 Helmholtz Center Munich, German Research Center for Environmental Health, Institute of Structural Biology, Neuherberg, Germany; 4 Technical University Munich, Center of Life and Food Sciences, Institute of Experimental Genetics, Freising-Weihenstephan, Germany; VIB & Katholieke Universiteit Leuven, Belgium

## Abstract

Fibroblast growth factor (Fgf) signalling plays a crucial role in many developmental processes. Among the Fgf pathway ligands, Fgf9 (UniProt: P54130) has been demonstrated to participate in maturation of various organs and tissues including skeleton, testes, lung, heart, and eye. Here we establish a novel *Fgf9* allele, discovered in a dominant *N*-ethyl-*N*-nitrosourea (ENU) screen for eye-size abnormalities using the optical low coherence interferometry technique. The underlying mouse mutant line *Aca12* was originally identified because of its significantly reduced lens thickness. Linkage studies located *Aca12* to chromosome 14 within a 3.6 Mb spanning interval containing the positional candidate genes *Fgf9* (MGI: 104723), *Gja3* (MGI: 95714), and *Ift88* (MGI: 98715). While no sequence differences were found in *Gja3* and *Ift88*, we identified an A→G missense mutation at cDNA position 770 of the *Fgf9* gene leading to an Y162C amino acid exchange. In contrast to previously described *Fgf9* mutants, *Fgf9^Y162C^* carriers were fully viable and did not reveal reduced body-size, male-to-female sexual reversal or skeletal malformations. The histological analysis of the retina as well as its basic functional characterization by electroretinography (ERG) did not show any abnormality. However, the analysis of head-tracking response of the *Fgf9^Y162C^* mutants in a virtual drum indicated a gene-dosage dependent vision loss of almost 50%. The smaller lenses in *Fgf9^Y162C^* suggested a role of Fgf9 during lens development. Histological investigations showed that lens growth retardation starts during embryogenesis and continues after birth. Young *Fgf9^Y162C^* lenses remained transparent but developed age-related cataracts. Taken together, *Fgf9^Y162C^* is a novel neomorphic allele that initiates microphakia and reduced vision without effects on organs and tissues outside the eye. Our data point to a role of Fgf9 signalling in primary and secondary lens fiber cell growth. The results underline the importance of allelic series to fully understand multiple functions of a gene.

## Introduction

According to the World Health Organization, 315 million people are visually impaired worldwide. 43 million of them suffer from complete blindness (<3/60 presenting visual acuity), leading to a significant loss of life quality and shortened life time. Globally, the major causes of blindness are (in order of frequency) cataracts, uncorrected refractive errors, glaucoma, and macular degeneration [1]. However, vision might also be impaired by irregular eye-size parameters as anterior chamber depth or thickness of the lens. Microphakia is characterized by smaller lenses and usually occurs together with other systemic abnormalities inside and outside the eye as part of the Warburg Micro syndrome (WARBM1; MIM #600118), Marfan's syndrome (MFS; MIM #154700), or rhizomelic *chondrodysplasia calcificans punctata* (RCDP; MIM #215100) [2]–[4]. These syndromes are initiated by mutations in *RAB3GAP* (WARMB1; MIM *602536), *FBN1* (MFS; MIM *134797), and *PEX7* (RCDP; MIM +601757), respectively [5]–[9].

Beside these structural and receptor proteins, various members of the Fgf family play a role in lens growth by regulating developmental processes. Fgfs are widely expressed in developing and adult tissues and have diverse functions in organogenesis, tissue repair, metabolism, and physiological homeostasis. In humans and mice, 22 Fgf ligands have been described, which mediate signals through seven different isoforms of Fgf receptors (FgfRs; reviewed by [10]). At least 13 Fgf ligands are present in the eye during development and/or at maturity (reviewed by [11]). Among them, Fgf1 (UniProt: P61148) and Fgf2 (UniProt: P15655) were initially demonstrated to regulate lens development by studies in lens epithelial explants [12]–[14]. In subsequent experiments, negative effects on lens polarity could be initiated by lens-specific over-expression of *Fgf1* (MGI: 95515), *Fgf3* (MGI: 95517), *Fgf4* (MGI: 95518), *Fgf7* (MGI: 95521), *Fgf8* (MGI: 99604), and *Fgf9* in the mouse [11], [15], [16].

Regarding null mutants of these ligands, only *Fgf9* knock-out mice displayed lens pathologies, characterized by delayed primary lens fiber cell elongation in at least a portion of *Fgf9* null embryos [17]. These *Fgf9* knock-out mutants further exhibited various pathologic phenotypes outside the eye including neonatal lethality caused by lung hypoplasia and male-to-female sex reversal initiated by irregular testicular embryogenesis [18]–[20]. Furthermore, irregular skeletogenesis was detected in an *Fgf9^N143T^* missense mutant (Fgf9*^Eks^*; MGI: 2182127). This is initiated by an impaired homodimerization of the altered Fgf9 protein, which increases diffusion through developing tissues due to decreased heparin binding affinity of Fgf9^EKS^ [21], [22].

In our attempt to identify novel genes involved in determination of eye size, we established the mouse mutant line *Aca12* (ACMaster abnormality #12) in a dominant ENU mutagenesis program [23] because of its significantly thinner lenses compared to data of control mice [24]. The goal of this study was the genetic and phenotypic characterization of *Aca12*. We identified a missense mutation in the fibroblast growth factor gene *Fgf9* as causative event for the observed microphakia. We further detected reduced vision in *Aca12* and demonstrate a retarded lens growth in *Aca12* embryos and postnatal mice. Our study indicates that the novel *Fgf9^Y162C^* allele has functional (and clinical) consequences different from other known alleles of *Fgf9*. Therefore, it shows the importance of the characterization of allelic series to fully understand the function of a given gene.

## Results

### Establishment and genotyping of the *Aca12* mouse mutant line

**Figure 1:**
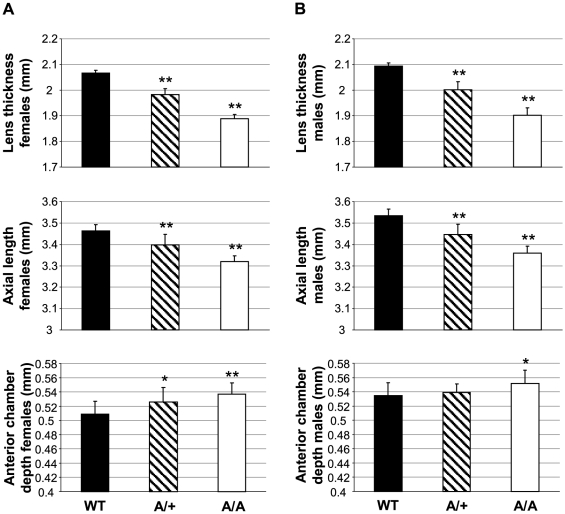
Affected eye size parameters of *Fgf9^Y162C^* mutants. (A,B) Mean lens thickness (polar diameter) and axial length were reduced in heterozygous (A/+, hatched) and homozygous *Fgf9^Y162C^* (A/A, white) mice compared to the C57BL/6J control (WT, black). Anterior chamber depth was increased in the mutants. (A) females (*n* = 20); (B) males (*n* = 20). *p<0.02; **p<0.001.

The mouse mutant line *Aca12* was originally detected and established in a dominant ENU mutagenesis screen for eye size abnormalities because of its decreased lens thickness (polar diameter) at the age of 11 weeks (10.1% and 9.4% reduced mean lens thickness in homozygous *Aca12* males and females, respectively). Further investigations revealed an increased anterior chamber depth and a reduced overall axial length. All eye-size parameters were less severely affected in heterozygotes, indicating a semidominant mode of inheritance (Figure 1A and 1B).

**Figure 2:**
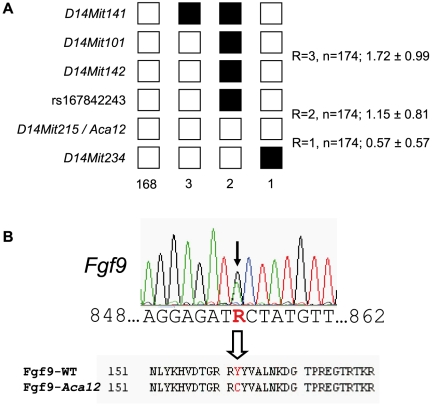
Haplotype analysis of the *Aca12* mutation and sequence analysis of the *Fgf9* coding region. (A) The *Aca12* mutation is localized between the flanking microsatellite markers *D14Mit268* and *D14Mit234*. Black boxes illustrate the presence of two C3H marker alleles (recombination between microsatellite marker and *Aca12*); white boxes illustrate heterozygosity of the markers for the C3H and C57BL/6J allele (lack of recombination). The number of G3 progeny carrying the particular recombination pattern is given below the boxes. The total number of recombination (R) events between neighbouring markers is shown to the right of the boxes, including the calculated relative genetic distances (cM ± standard deviations). In total, we tested 175 G3 animals. (B) Sequence analysis of genomic DNA of a heterozygous mutant shows an A→G transition (black arrow) at cDNA position 855 of the *Fgf9* gene resulting in a Y162C amino acid exchange in the Fgf9 protein.

In order to identify the mutation underlying the *Aca12* phenotype, we performed a genome-wide linkage analysis by crossing homozygous mutants on a C57BL/6J background (G1) to wild-type C3HeB/FeJ mice. Heterozygous mutants (G2) were backcrossed to C3HeB/FeJ mice. The SNP-based analysis of 55 G3 offspring revealed linkage of the *Aca12* phenotype to chromosome 14 within an interval spanning 13 Mb (between rs13482161 and rs30895903; cytoband C1 to C3). A further fine mapping using 175 G3 mice located the *Aca12* mutation close to the microsatellite marker *D14Mit215*, between rs167842243 and *D14Mit234* (Figure 2A). Based on these data, the genetic order was calculated (genetic distance ± SD in parenthesis): rs167842243 (1.15±0.81 cM), *Aca12*/*D14Mit215* (0.57±0.57 cM), *D14Mit234*. The critical interval of approximately 3.6 Mb includes 78 genes. 29 of them are predicted only because of the existence of ESTs, but are not yet fully annotated. Among the characterized genes, *Gja3* (gap junction protein, alpha 3), *Ift88* (intraflagellar transport 88 homolog [*Chlamydomonas*]), and *Fgf9* have previously been associated with irregularities in the eye [17], [25], [26]. Sequence analysis excluded *Gja3* and *Ift88* as candidates for *Aca12*. However, heterozygous *Aca12* carriers exhibited an A→G transition at cDNA-position 855 of *Fgf9* (counting the starting ATG as position 1; Figure 2B). Since the transition did not generate a novel restriction site, we confirmed the presence of this particular mutation in five homozygous carriers by sequencing of the corresponding region. Moreover, sequence analysis of four different wild-type strains (C57BL/6J, C3HeB/FeJ, JF1, and CFW) excluded a general polymorphism at this site. The 855 A→G transition is predicted to cause an exchange of Tyr to Cys at amino acid position 162 of the Fgf9 protein. Consequently, we refer to the novel allele as *Fgf9^Y162C^*.

### *Fgf9^Y162C^* does not affect viability, sex determination, or skeletogenesis

**Figure 3:**
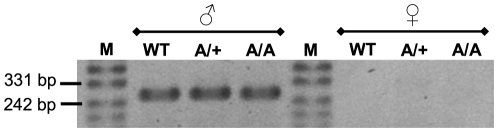
*Fgf9^Y162C^* mutants exhibit regular sexual development. Sex genotyping PCR amplified a *Sry* fragment (266 bp) indicating the presence of a Y chromosome in all tested male samples, but in none of the analyzed female probes. WT, C57BL/6J control (*n* = 5); A/+, heterozygous *Fgf9^Y162C^* mice (*n* = 6–9); A/A, homozygous *Fgf9^Y162C^* mice (*n* = 6–12); M, marker.

**Figure 4:**
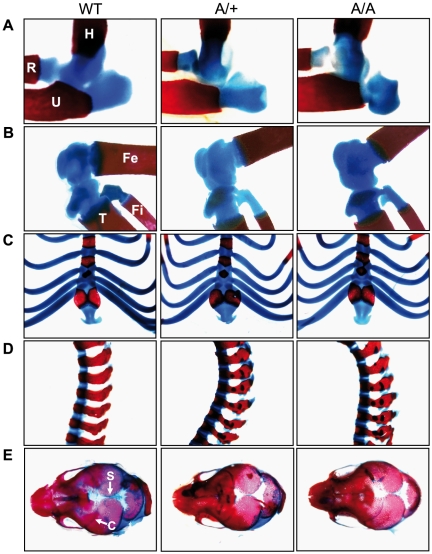
Skeleton develops regularly in *Fgf9^Y162C^* mutants. (A–E) Alcian blue/Alizarin red staining of (A) elbow joints (lateral view), (B) knee joints (lateral view), (C) thoraric regions (ventral view), (D) spinal columns (lateral view), and (E) skulls (dorsal view) of C57BL/6J control (WT), heterozygous *Fgf9^Y162C^* (A/+), and homozygous *Fgf9^Y162C^* (A/A) newborn mice. The mutants did not exhibit skeletal abnormalities. H, humerus; R, radius; U, ulna; Fe, femur; T, tibia; Fi, fibula; S, sagittal suture; C, coronal suture.

In order to study whether *Fgf9^Y162C^* carriers are fully viable, heterozygous individuals were bred to produce homozygous mutant mice. Among 86 intercross offspring, the genotype distribution was at the expected Mendelian ratio (25.6%, wild-types; 47.7%, heterozygotes; and 26.7%, homozygous mutants) and consequently did not indicate a reduced viability. Regular sex development in *Fgf9^Y162C^* was proved by sex genotyping PCR. A *Sry* signal was generated in all tested heterozygous and homozygous mutant males. In contrast, none of the female-like samples showed this signal, excluding the presence of XY genotyped female-like individuals (Figure 3). Concerning skeletogenesis, neither heterozygous nor homozygous *Fgf9^Y162C^* newborn mice developed elbow joint fusions, knee joint dysplasia, widened sternums, thickened ribs, fused vertebrae, or fused skull sutures (Figure 4A–4E). Moreover, even after breeding of more than 10 generations, we never observed kinky tails among adult *Fgf9^Y162C^* mice further demonstrating that skeletogenesis is not affected by the novel allele *Fgf9^Y162C^*.

### *Fgf9^Y162C^* mice show reduced vision that is not caused by retinal irregularities

**Figure 5:**
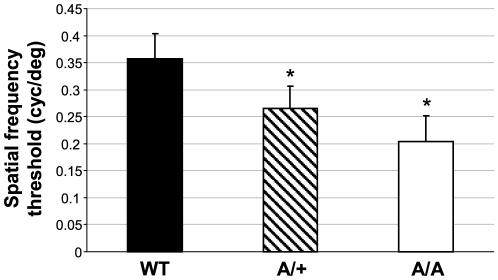
Reduced visual acuity in the *Fgf9^Y162C^* mutants. Spatial frequency thresholds indicated a reduced vision in the *Fgf9^Y162C^* mutants. Values represent means ± standard deviation of measurements from twelve C57BL/6J controls (WT), ten heterozygous *Fgf9^Y162C^* mutants (A/+), and ten homozygous *Fgf9^Y162C^* carriers (A/A). *p<0.001.

**Figure 6:**
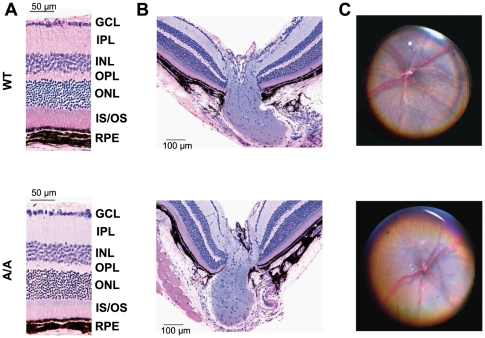
Regular retinal histology and fundus morphology of *Fgf9^Y162C^* mutants. (A,B) Morphology of four-month-old C57BL/6J control (WT) and homozygous *Fgf9^Y162C^* (A/A) retinas (A) and optic nerve heads (B) are compared. The mutants displayed a regular retinal development. (C) Funduscopy of six-month-old C57BL/6J controls (WT) and homozygous *Fgf9^Y162C^* (A/A) mice also revealed a regular pigmentation, optic disc structure, and blood vessel pattern in the eye fundi of the mutants. GCL, ganglion cell layer; IPL, inner plexiform layer; INL, inner nuclear layer; OPL, outer plexiform layer; ONL, outer nuclear layer; IS/OS, inner segment/outer segment of photoreceptor layer; RPE, retinal pigment epithelium.

**Figure 7:**
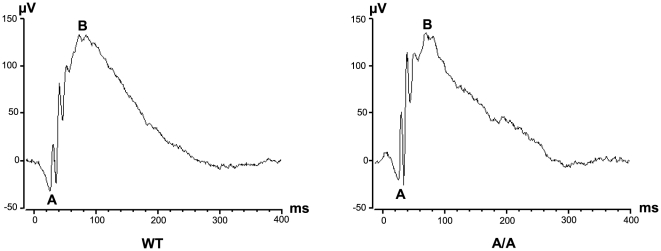
*Fgf9^Y162C^* mice exhibit regular functionality of the retina. Electroretinograms of six-month-old C57BL/6J control (WT) and homozygous *Fgf9^Y162^* ^C^ (A/A) mice are shown. The mutants exhibit a regular electrical response to the given light flashes. (A), a-wave amplitude; (B), b-wave amplitude.

Since malformations outside the eye were excluded, we focused our studies on visual properties and development of ocular tissues. Vision of *Fgf9^Y162C^* mice was tested in a virtual optokinetic drum with three-month-old carriers. Calculation of mean spatial frequency thresholds confirmed a negative effect on vision in both homozygotes and heterozygotes (Figure 5). One possible reason for the reduced vision might be an abnormal retinal morphology and functionality, especially since Fgf9 has been suggested to play a role in retinal differentiation and maturation [27]. We therefore analyzed the retina of the more severely affected homozygous *Fgf9^Y162C^* mice in detail. An initial histological comparison between *Fgf9^Y162C^* mutants and wild-type controls did not reveal abnormalities on the cellular level (Figure 6A and 6B). These findings were confirmed by ophthalmoscopic investigations. All tested mutants exhibited a homogenously pigmented fundus with a well defined vessel pattern and a regularly developed optic disc (Figure 6C). Further investigations of retinal function by ERG measurements showed standard implicit times and regular a- and b-wave amplitudes in the electroretinogram of homozygous *Fgf9^Y162C^* retinas (Figure 7). It clearly demonstrates that the function of retinal photoreceptors, bipolar neurons, and ganglions is not affected as far as can be identified with these methods.

### *Fgf9^Y162C^* retards prenatal and postnatal lens growth

**Figure 8:**
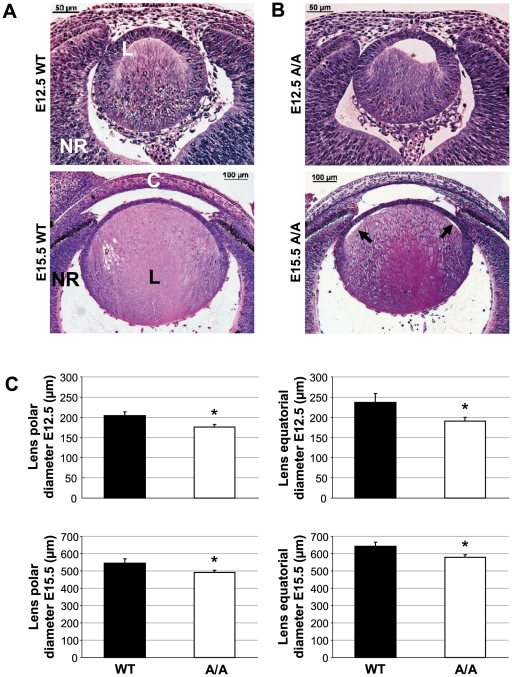
Retarded primary lens fiber growth and reduced size of the *Fgf9^Y162C^* prenatal lens. (A,B) Lens morphology of (A) C57BL/6J control (WT) and (B) homozygous *Fgf9^Y162C^* (A/A) lenses is shown at E12.5/E15.5. Growth of primary lens fibers is retarded in the mutants at E12.5. At E15.5, connection between lens fibers and lens epithelium is partly disrupted (arrows). L, lens; NR, neural retina; C, cornea. (C) Mean lens polar and equatorial diameter calculation at E12.5/E15.5 indicated a reduced size of both dimensions in the homozygous mutants. Values are means ± standard deviation (*n* = 5). *p<0.01.

**Figure 9:**
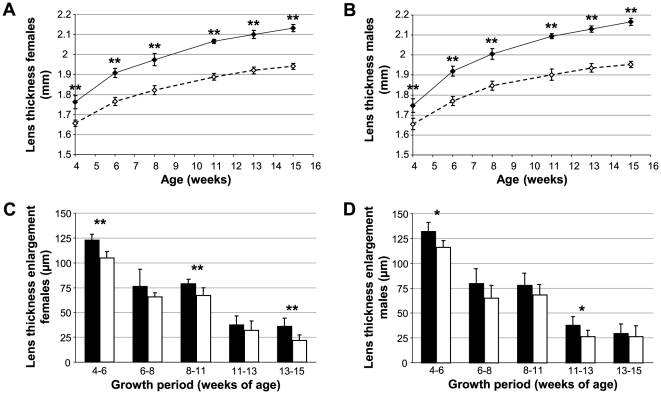
Decreased *Fgf9^Y162C^* lens thickness at postnatal stages and retarded postnatal lens growth in *Fgf9^Y162C^* mutants. (A,B) Mean lens thickness (polar diameter) of homozygous *Fgf9^Y162C^* mutants (dashed lines) obtained by OLCI at different postnatal stages are compared to the C57BL/6J control data (solid lines). Mutants exhibit significantly reduced lens sizes at all tested stages. (A) females; (B) males. (C,D) Postnatal lens growth of homozygous *Fgf9^Y162C^* mice (white bars) and C57BL/6J controls (black bars) is tracked by OLCI. In the mutants, lens growth is most strikingly reduced at early postnatal stages. (C) females; (D) males. Values are means ± standard deviation (*n* = 10–12). *p<0.01; **p<0.001.

We further used the novel *Fgf9^Y162C^* missense mutation to study the role of Fgf9 signalling during lens development. Comparable to the recent findings, lens vesicles of embryonic *Fgf9^Y162C^* lenses remained partly unfilled at E12.5, pointing to retarded primary lens fiber growth (Figure 8A and 8B). At E15.5, lens fiber cells elongated completely. However, connection between fibers and lens epithelium was partly disrupted in the mutants at this particular stage (Figure 8A and 8B). Further calculations of mean lens polar and equatorial diameters revealed a significant reduction of both dimensions in prenatal *Fgf9^Y162C^* lenses (Figure 8C). Postnatal OLCI measurements confirmed decreased polar diameters (Figure 9A and 9B). Moreover, postnatal growth tracking of individual *Fgf9^Y162C^* lenses indicated a significantly reduced lens growth rate in the tested growth periods (Figure 9C and 9D).

**Figure 10:**
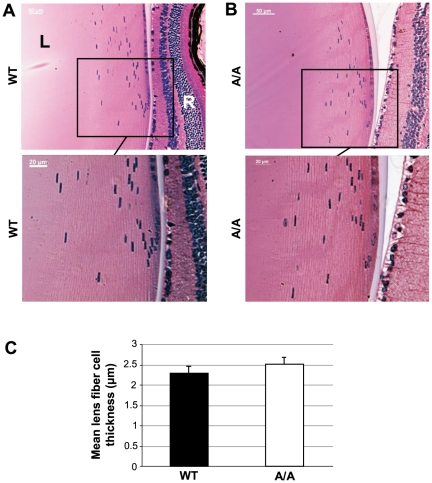
Regular equatorial lens histology and fiber cell size of *Fgf9^Y162C^* mutants. (A,B) Morphology of (A) four-month-old C57BL/6J control (WT) and (B) homozygous *Fgf9^Y162C^* (A/A) lenses is shown. The mutants display a regularly developed lens bow. L, lens; R, retina. (C) Mean lens fiber cell thickness at the age of four months calculated by averaging the breadth of ten fiber cell layers in the equatorial outer cortex. Lens fiber cell size did not differ significantly in the homozygous *Fgf9^Y162C^* mutants. Values are means ± standard deviation (*n* = 5).

**Figure 11:**
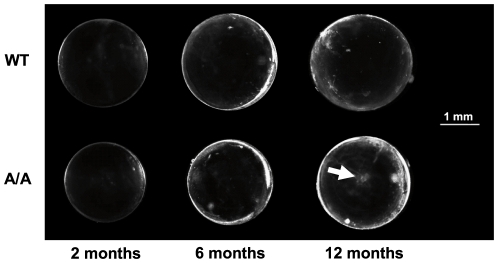
Age-related cataract formation in *Fgf9^Y162C^* lenses. C57BL/6J control (WT) and homozygous *Fgf9^Y162C^* (A/A) lenses are compared at three different stages (two, six, and twelve months of age). All lenses remained transparent at least up to six months of age. Some twelve-month-old mutant lenses developed an age-related polar cataract (arrow).

Reduced lens size did not affect transparency, at least at younger stages. Histological analysis of four-month-old lenses confirmed normal nuclear degradation as well as regular lens fiber cell arrangement and size (Figure 10A–10C). However, 11 out of 18 twelve-month-old mutant lenses exhibited faint opacities of fiber cells at the anterior pole (Figure 11), indicating a tendency towards age related cataract formation in *Fgf9^Y162C^* mice.

### *Fgf9^Y162C^* does not alter binding affinity for heparin

**Figure 12:**
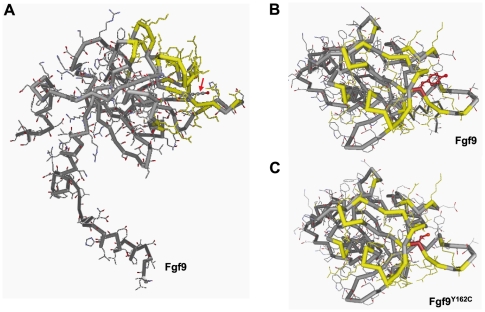
Predictive modeling of the Fgf9^Y162C^ protein. (A) The lateral view on Fgf9*^WT^* structure shows that the exchanged amino acid residue (red arrow) lies close to essential residues of the heparin binding region (marked in yellow). (B,C) Dorsal view on the wild-type protein (B) and Fgf9*^Y162C^* (C) indicates that structural changes within the heparin binding region (yellow marked residues) are not predicted. The amino acid residues at position 162 are marked in red.

**Figure 13:**
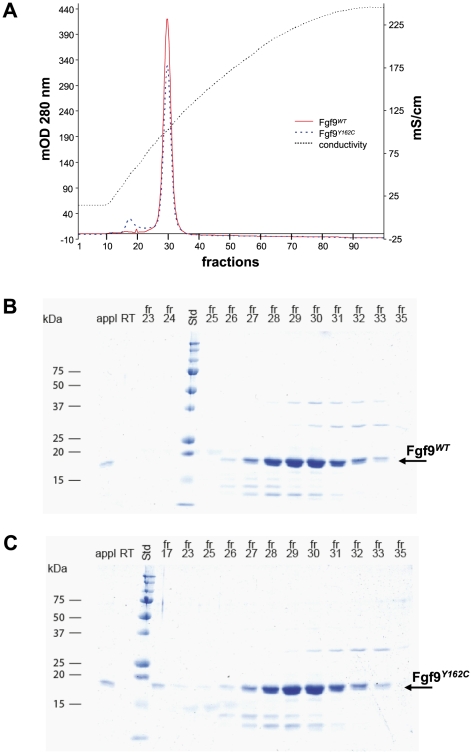
Affinity for Fgf9*^WT^* and Fgf9*^Y162C^* to heparin. (A) Chromatographic analysis of the affinities of mFgf9*^WT^* and mFgf9*^Y162C^* for heparin is shown. mFgf9*^WT^* and mFgf9*^Y162C^* were loaded onto a HiTrap heparin column (1.5 mg each) and eluted with a linear gradient of NaCl from 120 mM to 2.0 M (conductivity line). Elution profiles of mFgf9*^WT^* (red) and mFgf9*^Y162C^* (blue) were determined by monitoring absorbance at 280 nm. (B,C) Coomassie stained SDS-PAGE gels monitoring the chromatographic analysis for mFgf9*^WT^* (B) and mFgf9*^Y162C^* (C). Std, standard; appl, sample load; RT, run through; fr, fraction.

In order to get first insights into pathologic effects at the protein level, we performed modeling analysis of Fgf9*^Y162C^*. Structural changes were not predicted by the 3D-JIGSAW comparative modeling server (Figure 12A–12C). To test if the affinity towards heparin is influenced by the mutation Y162C, N-terminally truncated mFgf9*^WT^* and mFgf9*^Y162C^* were recombinantly expressed in bacteria and purified to approximately 95% purity. The proteins were subsequently applied to analytical heparin affinity chromatography. The major portions of both protein samples eluted exactly at the same ionic strength (Figure 13A). SDS PAGE (Figure 13B and 13C) as well as Western Blots (not shown) confirmed this observation. Thus, the mutation seems to have no effect on the heparin binding affinity.

## Discussion

We have established the novel mouse mutant *Aca12* that is primarily characterized by changes of ocular structures, e.g. larger anterior chambers, thinner lenses, and shorter axes. A genome wide linkage analysis and fine mapping placed the mutation on chromosome 14 within a 3.6 Mb interval. Sequence studies of positional candidate genes identified a base pair exchange within the *Fgf9* gene (chromosome position 58.69–58.73 Mb); this mutation co-segregates with the phenotype among our breeding colony and was not found within other wild-type strains of mice. The facts that (i) a recombination between the *Aca12* mutation and *D14Mit215* (chromosome position 58.99 Mb) did not occur among 175 G3 carriers of the linkage study, (ii) the 855 A→G transition co-segregates with the phenotype and does not occur as a polymorphic site in other mouse strains, and (iii) *Fgf9* has previously been associated with ocular malformations [17] further support the conclusion that the 855 A→G mutation represents the causative event for the observed pathologic phenotypes.

The replaced tyrosine residue 162 in Fgf9 is conserved between mice and humans and is an essential component of one of the 12 β strands defining the FGF trefoil fold; it is present also in FGF16 (UniProt: O43320) and FGF20 (UniProt: Q9NP95; they form together with FGF9 [UniProt: P31371] one of the FGF subfamilies [28]). Pathologic missense mutations in *FGF9* (MIM #600921)/*Fgf9* have been identified in humans affecting amino acid position 99 (S99N [29]) and position 143 in mice (N143T [22]). Among other features, in both cases, skeletal malformations have been described; however, effects on the skeleton have not been identified in the new *Fgf9^Y162C^* allele. Nevertheless, we demonstrate for the first time an essential role of this conserved residue Tyr-162 for the functional integrity of the Fgf protein. Position 162 is localized close to essential residues of the heparin binding site [30]. However, experimental data with recombinant proteins suggest no influence of the mutation on the heparin binding affinity.

Because of the unique phenotype, the novel *Fgf9^Y162C^* allele represents most likely a dominant-negative or neomorphic mutation, since the pathological phenotype is present in the heterozygotes. This feature is different from the recessive mode of inheritance in the *Fgf9* knockouts; these heterozygotes are phenotypically normal [18]. However, similar to our new *Fgf9^Y162C^* allele, the *Fgf9^Eks^* allele (characterized by a N143T substitution [22]) is also characterized by a dominant mode of inheritance [21]. In contrast to our new *Fgf9^Y162C^* allele, the *Fgf9^Eks^* allele leads to several skeletal defects. The molecular explanation includes the prevention of homodimerization of Fgf9*^Eks^*, a reduced binding affinity to heparin [22], and increased diffusion properties. Therefore, Fgf9*^Eks^* is present in regions, where it usually does not occur; this ectopic Fgf9 signalling represses skeletal joint and suture development [21]. In contrast to Fgf9*^Eks^*, the regular skeletogenesis, the viability of the homozygous mutants and the regular sex determination in *Fgf9^Y162C^* mutants strongly points to a different pathologic mechanism in these mutants.

To understand the role of homodimerization, Kalinina et al. [28] introduced structure-based mutations into the dimer interfaces of FGF9 (D195A, L200A, I204A, L205A). The corresponding mutant proteins occur more frequently as a monomer, exhibit enhanced proliferative activities, and bind heparin more weakly than wild-type Fgf. It would be of great interest to see whether the novel *Fgf9^Y162C^* allele leads to similar biochemical consequences.

Since the resulting pathologic symptoms are restricted to decreased eye sizes and reduced visual properties, we focused our detailed analysis on the ocular function. Since ERG and retinal histology were normal in the mutants, reduced vision is obviously not caused by irregular development of retinal layers or optic nerve head. Consequently, the altered ocular structures of the *Fgf9^Y162C^* mutants characterized by deeper anterior chambers (about 4%), thinner lenses (about 10%), and shorter axes (about 5%) might be responsible for the reduced vision. Moreover, reduced vision might be due also to irregular development of visual system in the brain, since *Fgf9* also is expressed in the visual cortex [31].

Our histological studies of embryonic *Fgf9^Y162C^* lenses indicated a retarded growth of primary lens fibers, since the lens vesicle of the mutants remained only partly filled at E12.5. This is in accordance with previous findings in *Fgf9* null embryos [17]. We additionally identified a dominant negative effect of Fgf9^Y162C^ on postnatal lens growth. Since secondary lens fiber cells are of regular shape and thickness, equatorial diameter of *Fgf9^Y162C^* lenses is most likely reduced because of a decreased total number of fiber cell layers. This would point to an essential role of Fgf9 signalling in proliferation of lens epithelial cells and fiber cell differentiation, respectively. Moreover, the reduced lens polar diameter might further indicate effects of *Fgf9^Y162C^* on lens fiber elongation. The decreased number of fiber cell layers does not affect transparency of the young lens, but about half of the tested lenses developed age-dependent polar opacifications.

Considering the confirmed role of Fgf9 signalling in lens development, it seems surprising that ocular *Fgf9* expression is restricted to retinal layers [17], [32], [33]. It therefore seems very likely that Fgf9 is secreted by retinal cells and diffuses through the vitreous to the lens. This would further explain the requirement of normal Fgf9 mobility for regular lens development. Taking into account that an increased diffusion of Fgf9*^Y162C^* can be excluded as discussed above, retarded lens growth might consequently be the result of reduced Fgf9*^Y162C^* concentrations in the lens tissue due to decreased diffusion abilities. This hypothesis is further supported by dose dependent effects of Fgf9 on lens development. In particular, transgenic overexpression of Fgf9 resulted in severe lens defects including irregular polarity and postnatal cataract formation [16]. Loss of Fgf9 function in homozygous knockout mutants initiated mild lens pathologies [17] which can be compared to the situation in heterozygous *Fgf9^Y162C^* mutants. However, completely different pathologic mechanisms (like altered receptor binding affinity) underlying the irregular lens development in *Fgf9^Y162C^* can currently not be excluded; polarity of lenses might develop normally because of an Fgf9*^Y162C^* concentration gradient. This hypothesis would fit to previous results that indicate an essential role of FGF gradients on determining anterior-posterior patterns of the lens [34], [35].

The target genes of Fgf9 signalling during lens development remain to be identified. Effects on lens fiber maturation have previously been described in various gap junction protein mutants including connexin23 (UniProt: Q9CX92), connexin43 (UniProt: P23242), connexin46 (UniProt: Q64448), and connexin50 (UniProt: P28236) [36]–[39]. Similar to the situation in homozygous *Fgf9^Y162C^* mice, irregular cell-to-cell appositions between lens epithelial cells and fiber cells were found in the connexin23- and connexin43 mutants [36], [37]. It seems very likely that Fgf9 signalling interacts with gap junction protein expression in the lens. Since Fgf9 binds specifically to the Fgf receptors 2 and 3 [40], studies using conditional knockout mice lacking Fgf receptor expression further provided hints for putative Fgf9 signalling targets. Triple knockout mice (*Fgfr1–3*; MGI: 95522–95524) exhibited irregular lens fiber elongation and proliferation together with aberrant or reduced expression of p27^kip1^ (UniProt: P46414), p57^kip2^ (UniProt: P49919), Prox1 (UniProt: P48437), Pax6 (UniProt: P63015), c-Maf (UniProt: P54843), E-cadherin (UniProt: P09803), and crystallins (UniProt: P24622, P02525, and P04345) [41].

In conlcusion, the *Fgf9^Y162^* mouse line described here provides a novel *Fgf9* allele with yet unknown properties. It will be useful for getting more insights into the role of Fgf9 signalling in lens development; moreover, our data suggest *FGF9* as a candidate for human age-related cataracts. Since the new *Fgf9* allele leads to a novel phenotype not observed in other of FGF9 mutants, it is of great interest to uncover the underlying biochemical differences.

## Materials and Methods

### Ethics statement

The use of animals was in accordance with the German Law of Animal Protection, the ARVO Statement for the Use of Animals in Ophthalmic and Vision Research, and the tenets of the Declaration of Helsinki. Animal experiments of this study were approved by the ethics committee of the Upper Bavarian government (TVA #55.2-1-54-2531-78-06).

### Mice

Mice were kept under specific pathogen-free conditions at the Helmholtz Center Munich. Male C57BL/6J mice were treated with ENU (80 mg/kg body weight applied by intraperitoneal injection in three weekly intervals) at the age of 10–12 weeks as previously described [42] and mated to untreated female C57BL/6J mice [23]. The offspring of the ENU-treated mice were screened at the age of 11 weeks for abnormalities of the eye size [24]. Mice with phenotypic deviations were tested for a dominant mode of inheritance.

### Linkage analysis

Heterozygous carriers (first generation) were mated to wild-type C3HeB/FeJ mice, and the offspring (second generation) were backcrossed to wild-type C3HeB/FeJ mice. DNA was prepared from tail tips of affected offspring of the third generation (G3). For genome-wide linkage analysis, genotyping of a genome-wide mapping panel consisting of 149 single nucleotide polymorphisms (SNP) was performed using MassExtend, a MALDI-TOF (matrix-assisted laser/desorption ionization, time of flight analyzer) mass spectrometry high-throughput genotyping system supplied by Sequenom (San Diego, CA, USA; [43]). Fine mapping was performed with the microsatellite markers *D14Mit141*, *D14Mit101*, *D14Mit142*, *D14Mit268*, *D14Mit215*, and *D14Mit234*. Furthermore, recombination frequency between *Aca12* and the SNP rs167842243 (chromosome 14, genome coordinates 56,682,854) was determined using the primer pair SNP56.68-1 and SNP56.68-2 (Table 1) and subsequent sequence analysis.

**Table 1:** PCR primers used in this study.

Short name	Sequence	Product size	Tm
SNP56.68-1	5′-GCAAATAAAGTTTGCATGACCA-3′		63.5°C
SNP56.68-2	5′-TTTGGAGCTGAGACGAAAGG-3′	398 bp	64.3°C
Fgf9-IV-1	5′-TTGAAGACTATTCTGGTTCAAAGA-3′		60.6°C
Fgf9-IV-2	5′-CAAAGTTTGGCAACAGTGGA-3′	471 bp	63.7°C
Sry-1	5′-GAGAGCATGGAGGGCCAT-3′		65.3°C
Sry-2	5′-CCACTCCTCTGTGACACT-3′	266 bp	55.8°C
Fgf9-I-1	5′-TCGCCTAGTGTCTCCTGGTT-3′		63.7°C
Fgf9-I-2	5′-GACCAGGCCCACTGCTATAC-3′	398 bp	63.4°C
Fgf9-II-1	5′-CGGTACTATCCAGGGAACCA-3′		63.6°C
Fgf9-II-2	5′-CAACAGTGGAGCTGAGGTGA-3′	497 bp	64.2°C
Gja3-I-1	5′-GGAATCCAGCACTGTCAGGT-3′		54.0°C
Gja3-I-2	5′-GCATGAAGATGACAAAGATGG-3′	700 bp	50.0°C
Gja3-II-1	5′-AGGCCCACAGAGAAGACCAT-3′		54.0°C
Gja3-II-2	5′-GGAATCCAGCACTGTCAGGT-3′	791 bp	54.0°C
Ift88-I-1	5′-ATCAGGCGTCGCTTCTTC-3′		63.1°C
Ift88-I-2	5′-TGATGTCAGGGATGTCTTGG-3′	297 bp	63.6°C
Ift88-II-1	5′-ATCAGGCGTCGCTTCTTC-3′		63.1°C
Ift88-II-2	5′-CTTTCTTCTCCAACTGTCTAATTTTT-3′	499 bp	60.8°C
Ift88-III-1	5′-ATTTGACCCCCTTGGTCAGT-3′		64.4°C
Ift88-III-2	5′-TTTATGGACACTTGGGATCTGA-3′	469 bp	63.1°C
Ift88-IV-1	5′-GCAATGCAGGAAGACTGAAA-3′		62.9°C
Ift88-IV-2	5′-CAAAAGACGCTTCGATCACA-3′	498 bp	63.9°C
Ift88-V-1	5′-AAGGAAAGCCATGGCAGAA-3′		64.4°C
Ift88-V-2	5′-TCCAGACGGTTCAGCTTCTT-3′	495 bp	63.9°C
Ift88-VI-1	5′-AGAGGCCCTGAGAAACGACT-3′		64.2°C
Ift88-VI-2	5′-AGCTACCATCAGCTGCCACT-3′	460 bp	63.9°C
Ift88-VII-1	5′-TGCGAGAAAGCCATTCAGTA-3′		63.4°C
Ift88-VII-2	5′-TTCTATCTGAGGGCCCAGTG-3′	486 bp	64.1°C
Ift88-VIII-1	5′-CAAAGAAATAGATGCCTCCTACG-3′		62.6°C
Ift88-VIII-2	5′-AAGTTCCAGGCCAGAGGAA-3′	250 bp	63.7°C
mFgf9for-33aa_NcoI	5′-TTTCCATGGATGGACCACCTGGGTCAG-3′		47.4°C
mFgf9rev_HindIII	5′-TTTAAGCTTTCAGCTTTGGCTTAGAATATC-3′	474 bp	48.5°C

### Genotyping, sex determination, and sequencing

Genomic DNA was isolated from tail tips of C57BL/6J, C3HeB/FeJ, JF1, and CFW wild-type mice or homozygous/heterozygous mutants according to standard procedures.

PCR of the mutated site was performed with the primers Fgf9-IV-1 and Fgf9-IV-2, sex genotyping PCR was carried out with the primers Sry-1 and Sry-2 amplifying the sex determining region of chromosome Y (Table 1). For sequencing of promising candidate genes, the primer pairs Fgf9-I/Fgf9-II, Gja3-I/Gja3-II, and Ift88-I to Ift88-VIII were used (Table 1).

PCR was performed with a PTC-225 thermocycler (MJ Research, Waltham, USA). Products were analyzed by electrophoresis on a 1.5% agarose gel. Sequencing was performed commercially (GATC Biotech, Konstanz, Germany) after direct purification of the PCR products (Nucleospin Extract II, Macherey-Nagel, Düren, Germany).

The coding sequence of the novel *Fgf9^Y162C^* allele is available under GenBank accession HM988990.

### Protein modeling

The PDB files used to model Fgf9*^WT^* and Fgf9*^Y162C^* was generated from the 3D-JIGSAW comparative modeling server [44]. The proteins were modeled using the 3D Mol-Viewer component of the Vector NTI Suite software 9.0.0 (Invitrogen, Darmstadt, Germany).

### Fgf9 bacterial expression, purification and heparin binding

Fgf9*^WT^* and Fgf9*^Y162C^* were expressed and purified essentially as previously described [22], [45]. Residues 34 to 208 each of mFgf9*^WT^* and mFgf9*^Y162C^* were cloned into the vector pET28a(+) (Novagen, Darmstadt, Germany) between *NcoI* and *HindIII* sites. Forward primer mFgf9for-33a_NcoI and reverse primer mFgf9rev_HindIII were used (Table 1). The resulting open reading frames coded for proteins of ∼20 kDa. Sequence integrity was verified by Sanger sequencing. Plasmid constructs were transformed into *E.coli* BL21 DE3 (Stratagene, Waldbronn, Germany) and protein expression was induced by 0.5 mM IPTG in LB/Kan medium for four hours at 37°C or overnight at 20°C. Bacteria were harvested, suspended in lysis buffer (25 mM HEPES pH 7.5, 150 mM NaCl, 10% glycerol, 1 mM EDTA, protease inhibitor Complete [Roche, Mannheim, Germany]) and subsequently disrupted by lysozyme treatment and sonication. DNA was digested by DNaseI (New England Biolabs, Frankfurt, Germany). After removal of cell debris by centrifugation, ammonium sulfate was added to the supernatants to reach 25% saturation. After centrifugation Fgf9 proteins were precipitated over night from the supernatant at 50% saturation of ammonium sulfate. Pellets were dissolved in 40 volumes 25 mM HEPES pH 7.5, 120 mM NaCl, centrifuged, and the supernatants applied to a 1 ml HiTrap heparin affinity column (GE Healthcare, Little Chalfont, United Kingdom). Proteins were eluted with a linear gradient (120 mM to 2 M NaCl) in HEPES pH 7.5. Fractions containing enriched mFgf9 proteins were applied to a second round of heparin affinity chromatography. Identity of mFgf9 proteins was verified by SDS-PAGE and Western blotting (polyclonal antibodies from Antibodies-Online, Aachen, Germany). Protein concentration of purified proteins was determined by Bradford assay (Bio-Rad, München, Germany) using BSA as standard.

### Analytical heparin affinity chromatography

1.5 mg each of ∼95% pure mFgf9*^WT^* and mFgf9*^Y162C^* were loaded onto a 1 ml HiTrap heparin HP column (GE Healthcare, Little Chalfont, United Kingdom) equilibrated with 25 mM HEPES pH 7.5, containing 120 mM NaCl. The bound proteins were eluted with a linear gradient of NaCl (120 mM to 2.0 M) in the same buffer.

### Skeleton preparation

Newborn mice were eviscerated and the skin was removed. After fixation in 100% ethanol (four days) and incubation in acetone (three days), mice were washed and stained in Alcian blue/Alizarin red solution (150 mg Alcian blue solved in 50 ml 70% ethanol, 50 mg Alizarin red solved in 50 ml 95% ethanol, 50 ml acetic acid, 850 ml 100% ethanol) for ten days. After staining, mice were washed, cleared in 1% KOH/20% glycerol (two weeks), and stored in 100% glycerol.

### Histological preparation, embryonic lens area determination, and mean lens fiber cell thickness calculation

Eyes of embryos and adult mice were histologically analyzed for retinal irregularities, lens area, and lens fiber cell pathologies. Embryo heads or separated eyes were fixed for seven days in Davidson solution and embedded in JB-4 plastic medium (Polyscience Inc. Eppelheim, Germany) according to the manufacturer's protocol. Sectioning was performed with an ultramicrotome (OMU3; Reichert-Jung, Walldorf, Germany). Serial transverse 3-µm sections were cut with a glass knife and stained with methylene blue and basic fuchsin. The sections were evaluated with a light microsocope (Axioplan, Carl Zeiss, Jena, Germany). Images were acquired by means of a scanning camera (AxioCam; Jenoptik, Jena, Germany) and imported into an image-processing program (Photoshop 10.0, Adobe, Unterschleissheim, Germany). Embryonic lens diameters and lens fiber cell sizes were analyzed using the size determination tools provided by the AxioVision 4.6.3.0 software (Carl Zeiss, Jena, Germany). Mean lens fiber cell thickness was calculated by averaging the breadth of ten fiber cell layers in the equatorial outer cortex.

### Lens growth determination and eye size measurement

The sizes of ocular parameters were examined between four and 15 weeks of age using optical low coherence interferometry (OLCI; “ACMaster”, Meditec, Carl Zeiss, Jena, Germany). Briefly, mice were anesthetized with an intraperitoneal injection of 137 mg ketamine and 6.6 mg xylazine per kilogram body weight. The anesthetized mouse was placed on a platform and oriented in an appropriate position using light signals from six infrared LEDs arranged in a circle that must be placed in the center of the pupil. Central measurements of lens thickness (polar diameter), axial length, corneal thickness, and anterior chamber depth as well as data evaluation were performed essentially as described [24], [46].

### Virtual vision test

Vision tests were performed between 9 am and 4 pm using a virtual optomotor system (Cerebral Mechanics, Lethbridge, Canada) as described previously [47]. Briefly, a rotating cylinder covered with a vertical sine wave grating was calculated and drawn in virtual three-dimensional space on four computer monitors facing to form a square. Visually unimpaired mice track the grating with reflexive head and neck movements (head-tracking). Vision threshold of the tested mice was quantified by a simple staircase test. Rotation speed and contrast were set to 12.0 d/s and 100%, respectively. Since no significant threshold differences were observed between males and females (*P*>0.05; calculated by Mann-Whitney U-test), data of both sexes were combined. Thresholds of wild-type C57BL/6J and homozygous *Fgf9^Y162C^* mice were compared using the Mann-Whitney U-test.

### Funduscopy

Nine-month-old mice with clear lenses were administered 1% atropine to each eye to dilate their pupils. The fundus examination was performed with a Heine Sigma 150K indirect ophthalmosocope (Haag-Streit GmbH, Wedel, Germany) and a Volk 90D superfield lens (Haag-Streit GmbH). Digital fundus images were taken with a Heine Video Omega 2C indirect ophthalmoscope connected to a VRmAVC Video Grabber (Dieter Mann GmbH, Mainaschaff, Germany) and a Volk 40D or 60D lens (Fronhäuser GmbH, Unterhaching, Germany). The images were imported in an image-processing program (Photoshop 10.0; Adobe).

### Elektroretinography (ERG)

Ganzfeld ERGs were recorded simultaneously from both eyes of six-month-old mice to examine the retinal function as described [48]. In brief, mice were dark-adapted for at least 12 hours and anaesthetized. After pupil dilation (1 drop Atropine 1%), individual mice were fixed on a sled and gold wires (as active electrodes) were placed on the cornea. The ground electrode was a subcutaneous needle in the tail; a reference electrode was placed subcutaneously between the eyes. The mice were introduced into an ESPION ColorBurst Handheld Ganzfeld LED stimulator (Diagnosys LLC, Littleton, MA, USA) on a rail to guide the sled (High-Throughput Mouse-ERG, STZ for Biomedical Optics and Function Testing, Tübingen, Germany). 10 ms light pulses were delivered at a frequency of 0.48 Hz and a light intensity of 12,500 cd/m^2^. Responses were recorded with an ESPION Console (Diagnosys LLC, Littleton, MA, USA).

### General

Chemicals were from Merck (Darmstadt, Germany) or Sigma Chemicals (Deisenhofen, Germany).
